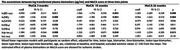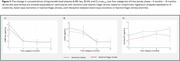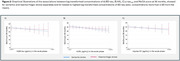# Associations between neurodegenerative and acute injury biomarkers and post‐stroke cognitive impairment – the Nor‐COAST study

**DOI:** 10.1002/alz70856_101515

**Published:** 2025-12-24

**Authors:** Anne‐Brita Knapskog, Guglielmo Di Molfetta, Heidi Vihovde Sandvig, Kaj Blennow, Henrik Zetterberg, Stina Vihovde Aam, Ingvild Vihovde Saltvedt, Nicholas Ashton

**Affiliations:** ^1^ Oslo University Hospital, Oslo, Norway; ^2^ Department of Psychiatry and Neurochemistry, Institute of Neuroscience and Physiology, The Sahlgrenska Academy, University of Gothenburg, Mölndal, Sweden; ^3^ Oslo University Hospital, OSLO, Norway; ^4^ Department of Psychiatry and Neurochemistry, Institute of Neuroscience & Physiology, the Sahlgrenska Academy at the University of Gothenburg, Mölndal, Gothenburg, Sweden; ^5^ Department of Psychiatry and Neurochemistry, Institute of Neuroscience and Physiology, the Sahlgrenska Academy at the University of Gothenburg, Mölndal, Sweden; ^6^ St. Olavs Hospital, TRONDHEIM, Norway; ^7^ Norwegian University of Science and Technology, Trondheim, Norway; ^8^ Department of Old Age Psychiatry, Institute of Psychiatry, Psychology & Neuroscience, King's College London, London, United Kingdom

## Abstract

**Background:**

Patients with vascular risk factors have a higher incidence of Alzheimer's disease (AD), and AD patients have an increased risk of experiencing stroke. About half of all stroke survivors experience post‐stroke cognitive impairment (PSCI). Stroke triggers numerous inflammatory and neurodegenerative processes both in the acute and chronic phases. The aims of this study were to investigate longitudinal plasma biomarker data in a stroke population and whether high levels of neurodegenerative and acute injury biomarkers at baseline could predict PSCI.

**Method:**

In this Nor‐COAST substudy, we included 547 stroke patients (Age [mean = 73± 12], 56 % males, 89 % ischemic strokes, 11 % haemorrhagic strokes). Brain‐derived tau (BD‐tau), phosphorylated tau_181_ (*p*‐tau_181_), total tau (t‐tau), amyloid β40 (Aβ_40_), Aβ_42_, glial fibrillary acidic protein (GFAP) and neurofilament light chain (NfL) concentrations were measured on the Simoa platform in plasma samples collected at inclusion, and after 3, 18 and 36 months. We explored the biomarkers’ associations with cognitive decline as measured by the Montreal Cognitive Assessment (MoCA) scale up to 36 months post‐stroke.

**Result:**

Within the first three months BD‐tau, t‐tau, and GFAP concentrations declined by 43%, 20% and 76% respectively, whereas NfL levels continued to decrease up to 18 months (59%). *p*‐tau_181_ increased gradually by 24 % from acute phase to 36 months. Aβ_42_ remained unchanged. Higher acute phase concentrations of BD‐tau, NfL and *p*‐tau_181_ predicted lover MoCA score up to 36 months post‐stroke in patients with ischemic stroke (*p* value < 0.05). However, including clinical parameters attenuated the associations. These associations were not found in the patients with haemorrhagic stroke.

**Conclusion:**

Concentrations of plasma BD‐tau, NfL, GFAP, and t‐tau were increased in the acute phase of stroke, but no significant change was seen for Aβ_42_. BD‐tau, t‐tau and GFAP concentrations decreased to stable levels within 3 months of event, NfL stabilized within 18 months, whereas *p*‐tau181 showed an increase. Higher concentrations of BD‐tau, NfL, and *p*‐tau_181_ were associated with worse cognitive outcome up to 36 months post‐stroke. These results indicate that stroke trigger a continuing neurodegenerative process, as demonstrated by gradually increasing concentrations of *p*‐tau_181_ leading to PSCI.